# Metabolomic data of phenolic compounds from *Acer negundo* extracts

**DOI:** 10.1016/j.dib.2020.105569

**Published:** 2020-04-21

**Authors:** Hebert Jair Barrales-Cureño, Rafael Salgado-Garciglia, Luis Germán López-Valdez, Juan Luis Monribot-Villanueva, José Antonio Guerrero-Analco, Gonzalo Guillermo Lucho-Constantino, Fabiola Zaragoza-Martínez, Braulio Edgar Herrera-Cabrera, César Reyes

**Affiliations:** a Instituto de Investigaciones Químico-Biológicas, Universidad Michoacana de San Nicolás de Hidalgo, Edificio B-3, Ciudad Universitaria, 58030, Morelia, Michoacán, México; b Laboratorio de Productos Naturales, Área de Química. AP74 Oficina de correos Chapingo, Universidad Autónoma Chapingo, Km 38.5 Carretera México-Texcoco, 56230, Texcoco, Estado de México, México; c Red de Estudios Moleculares Avanzados, Clúster BioMimic®, Instituto de Ecología, A.C., Xalapa, Ver., México; d Universidad Tecnológica de Gutierrez Zamora, Veracruz. Prolongación Dr. Miguel Patiño S/N, Centro, 93556, Veracruz, México; e Centro de Investigación y Estudios Avanzados del IPN. Av Instituto Politécnico Nacional 2508, San Pedro Zacatenco, Gustavo A. Madero, 07360, Ciudad de México, México; f Colegio de Postgraduados, Campus Puebla. Km. 125.5, Carr. Federal México-Puebla, Santiago Momoxpan, 72760, Puebla, México; g Universidad Intercultural del Estado de Puebla, Calle Principal a Lipuntahuaca S/N, 73475, Puebla, México

**Keywords:** Antioxidant, flavonoids, gentisic acid, kaempferol-3-O-glucoside, quercetin-3-glucoside

## Abstract

Phytochemical and metabolomic data were obtained for the most important phenolic compounds in ethanolic extracts from the endangered *Acer negundo* tree in Morelia, Michoacan. Samples of leaves and stems were subjected to ethanolic extraction with electric rotavapor. We developed a metabolomic analysis that encompassed the correlation between the leaf and stem extracts through principal component analysis. The data were obtained with an infinity Agilent ultrahigh resolution liquid chromatograph coupled to a Agilent triple quadrupole mass spectrometer. The protocol used was a dynamic MRM (Multiple Reaction Monitoring). Clustering result shown as heatmap (distance measure using euclidean, and clustering algorithm using ward.D).

Specifications Table

**Table:** 

**Subject**	Botany, Phytochemistry, Plant biotechnology, Metabolomics, Food chemistry, Chemistry of natural products.
**Specific subject area**	Metabolomic analysis, liquid chromatography, mass spectrometry.
**Type of data**	Table, Figure, Image
**How data were acquired**	Leaf sample collection, stem sample collection, liquid chromatography, mass spectrometry.
**Data format**	Raw and Analysed
**Parameters for data collection**	Samples of leaves and stems of *Acer negundo* tree were collected and subjected to a dehydration process that required three days at a temperature of 50 °C in rotary evaporator.
**Description of data collection**	A botanical exploration of the samples was conducted to obtain the ethanolic extracts. The samples were filtered and then the solvent was evaporizated in an electric rotavapor to obtain the crude extracts. The samples were collected for metabolomic analysis by liquid chromatography and mass spectrometry. Identification and quantification of the analyzed phenolic compounds in leaf and stem extracts were obtained. A heat map was obtained. The equipment used was a UPLC coupled to a triple quadrupole mass spectrometer. The equipment was injected with 2 µL of ethanolic extract from leaves and 2 µL of ethanolic extract from stems.
**Data source location**	Morelia, Michoacán, México Country: México The GPS coordinates are Latitude and longitude for collected samples/data: West, 1920 m.a.s.l.
**Data accessibility**	Repository name: Mendeley Data Data identification number: 2 Direct URL to data: http://dx.doi.org/10.17632/hhp8z52n9t.2

## Value of the data

• The data serve to identify and quantify the type and concentration of the metabolites present in the plant organs of *Acer negundo.*
• The data collected could increase the knowledge about the level of phenolic compounds in endangered trees such as *Acer negundo.*
• The distribution of the quantitative data could serve as a reference for metabolomic studies in other species of the genus *Acer negundo.*
• The data of quantification of metabolites type phenolic compounds with antioxidant power in food chemistry allows the standardization of quality products from *Acer negundo.*
• A correlation of metabolites and a database of metabolites of this species is obtained for metabolomics studies in trees of medical importance.
• Currently, no metabolomic studies have been conducted on this species, and therefore it is important for studies in biochemistry, biosynthesis, plant physiology, plant biotechnology, phytochemistry and food chemistry.

## 1 Data Description

**Figure 1:**
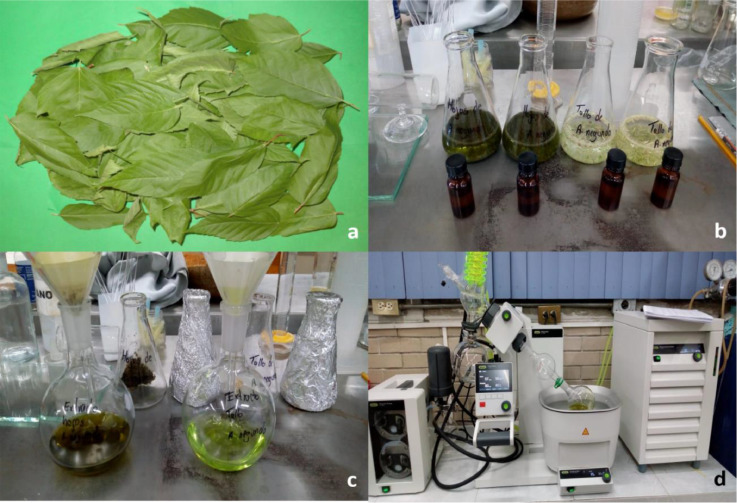
a) Biological sample of *Acer negundo* leaves and stems; b) Incorporation of the solvent c) Filtration of the samples, and d) Rotaevaporization of the solvent to obtain the raw extract.

The data set in this article describes the metabolomics that includes all phenolic compounds synthesized in the leaves and stems of the *Acer negundo* tree. Fig. 1 describes the extraction process obtained from our protocol in which 80% ethanolic solution is used and the raw extract is obtained from a rotavaporizer. Currently, metabolomics allows the identification and quantification of total metabolites in a plant cell, or plant tissue [1], [2].

The protocol used was a dynamic MRM (Multiple Reaction Monitoring). The conditions for each compound are described in the Table 1. The retention time variation allowed for the search of the compounds were 2 min in each case. The cell accelerator voltage was 7 V for each compound. Dilutions were made if the concentration of some compounds were higher than the linearity range.

**Table 1:** Conditions for the quantification of mass spectrometry data.

Compound	dMRM transition			Mass spectrometric conditions			Quantification conditions		
	Precursor ion	Product ion	Retention time	Collision energy	Fragmentor	Polarity	Quantification range (µM)	Regression type	R^2^
Shikimic acid	173.1	111.1	0.48	10	100	Negative	0.25 - 18	Quadratic	0.99
Gallic acid	169.0	125.2	1.17	10	100	Negative	0.25 - 18	Quadratic	0.99
L-Phenylalanine	166.1	131.0	1.85	10	100	Positive	0.25 - 18	Quadratic	0.99
Protocatechuic acid	153.0	109.1	2.23	10	100	Negative	0.25 - 18	Quadratic	0.99
4-Hydroxybenzoic acid	137.1	92.8	3.43	10	100	Negative	0.25 - 18	Quadratic	0.99
Gentisic acid	153.0	109.0	3.43	10	100	Negative	0.25 - 18	Quadratic	0.99
(-)-Epigallocatechin	305.1	125.0	4.27	20	140	Negative	0.25 - 18	Quadratic	0.99
4-Hydroxyphenylacetic acid	107.1	77.0	4.5	20	140	Positive	0.25 - 18	Quadratic	0.99
(+)-Catechin	291.0	138.9	4.58	10	100	Positive	0.25 - 18	Quadratic	0.99
Vanillic acid	169.0	93.0	4.75	10	100	Positive	0.25 - 18	Quadratic	0.99
Scopolin	355.1	193.0	4.83	20	100	Positive	0.25 - 18	Quadratic	0.99
Caffeic acid	181.0	163.0	4.90	10	100	Positive	0.25 - 18	Quadratic	0.99
Chlorogenic acid	355.1	163.0	4.90	10	100	Positive	0.25 - 18	Quadratic	0.99
Malvin	655.1	331.1	5.22	40	100	Positive	0.25 - 18	Quadratic	0.99
Kuromanin	449.0	286.9	5.6	30	100	Positive	0.25 - 18	Quadratic	0.99
Procyanidin B2	577.1	425.1	5.89	10	100	Negative	0.25 - 18	Quadratic	0.99
Vanillin	153.0	124.9	6.16	10	100	Positive	0.25 - 18	Quadratic	0.99
Keracyanin	595.2	287.1	6.18	20	100	Positive	0.25 - 18	Quadratic	0.99
(-)-Epicatechin	291.0	138.8	6.44	10	100	Positive	0.25 - 18	Quadratic	0.99
Mangiferin	423.0	302.0	6.64	10	100	Positive	0.25 - 18	Quadratic	0.99
4-Coumaric acid	165.0	147.0	6.69	10	100	Positive	0.25 - 18	Quadratic	0.99
Umbelliferone	163.0	107.0	7.16	30	100	Positive	0.25 - 18	Quadratic	0.99
(-)-Gallocatechin gallate	458.9	139.0	7.29	20	80	Positive	0.25 - 18	Quadratic	0.99
Scopoletin	193.0	133.0	7.86	10	100	Positive	0.25 - 18	Quadratic	0.99
Ferulic acid	195.1	145.0	8.1	20	100	Positive	0.25 - 18	Quadratic	0.99
Quercetin 3,4-di-O-glucoside	627.0	302.9	8.18	10	100	Positive	0.25 - 18	Quadratic	0.99
3-Coumaric acid	165.0	147.0	8.49	10	100	Positive	0.25 - 18	Quadratic	0.99
Sinapic acid	225.1	207.1	8.58	10	100	Positive	0.25 - 18	Quadratic	0.99
Salicylic acid	137.0	93	8.97	10	100	Negative	0.25 - 18	Quadratic	0.99
Ellagic acid	300.5	145.0	9.0	30	170	Negative	0.25 - 18	Quadratic	0.99
Epicatechin gallate	443.1	123.0	9.36	10	100	Positive	0.25 - 18	Quadratic	0.99
Myricitrin	465.0	318.9	9.38	10	100	Positive	0.25 - 18	Quadratic	0.99
Quercetin 3-D-galactoside	465.0	302.9	9.58	10	100	Positive	0.25 - 18	Quadratic	0.99
Rutin	611.0	302.9	9.74	10	100	Positive	0.25 - 18	Quadratic	0.99
Quercetin 3-glucoside	465.0	303.0	9.91	10	100	Positive	0.25 - 18	Quadratic	0.99
Luteolin 7-O-glucoside	449.0	287.0	10.24	10	100	Positive	0.25 - 18	Quadratic	0.99
*p*-Anisic acid	153.1	109.0	10.26	5	120	Positive	0.25 - 18	Quadratic	0.99
2,4-Dimethoxy-6-methylbenzoic acid	197.0	179.0	11.11	5	80	Positive	0.25 - 18	Quadratic	0.99
Penta-O-galloyl-B-D-glucose	771.1	153.0	11.23	20	100	Positive	0.25 - 18	Quadratic	0.99
Kaemperol 3-O-glucoside	449.0	286.9	11.27	10	100	Positive	0.25 - 18	Quadratic	0.99
Quercitrin	449.1	303.1	11.34	10	100	Positive	0.25 - 18	Quadratic	0.99
Myricetin	317.0	179.0	11.49	10	100	Negative	0.25 - 18	Quadratic	0.99
Naringin	273.0	153.0	11.89	10	120	Positive	0.25 - 18	Quadratic	0.99
*trans*-Resveratrol	229.1	135.1	11.94	10	100	Positive	0.25 - 18	Quadratic	0.99
Rosmarinic acid	361.1	163.0	12.35	10	100	Positive	0.25 - 18	Quadratic	0.99
Hesperidin	609.1	301.1	12.48	20	100	Negative	0.25 - 18	Quadratic	0.99
Secoisolariciresinol	363.2	137.1	12.58	20	100	Positive	0.25 - 18	Quadratic	0.99
Phloridzin	435.0	272.9	12.81	10	100	Negative	0.25 - 18	Quadratic	0.99
*trans*-Cinnamic acid	149.1	131.0	13.93	10	100	Positive	0.25 - 18	Quadratic	0.99
Psoralen	187.0	131.1	14.24	20	100	Positive	0.25 - 18	Quadratic	0.99
Quercetin	302.9	153.1	14.47	35	100	Positive	0.25 - 18	Quadratic	0.99
Luteolin	287.1	153.0	14.56	30	100	Positive	0.25 - 18	Quadratic	0.99
Cirsimarin	477.0	314.9	14.93	10	100	Positive	0.25 - 18	Quadratic	0.99
Angelicin	187.0	131.1	15.03	20	100	Positive	0.25 - 18	Quadratic	0.99
Naringenin	271.0	151	16.2	10	100	Negative	0.25 - 18	Quadratic	0.99
Apigenin	271.0	153.0	16.72	30	100	Positive	0.25 - 18	Quadratic	0.99
Citropten	207.0	192.0	16.92	20	100	Positive	0.25 - 18	Quadratic	0.99
Matairesinol	359.2	137.1	17.02	10	100	Positive	0.25 - 18	Quadratic	0.99
Kaempferol	287.1	153.0	17.09	30	100	Positive	0.25 - 18	Quadratic	0.99
Hesperetin	303.1	177.1	17.5	20	100	Positive	0.25 - 18	Quadratic	0.99
Podophyllotoxin	415.1	397.1	18.68	10	100	Positive	0.25 - 18	Quadratic	0.99
Methyl cinnamate	163.1	131.0	20.92	6	100	Positive	0.25 - 18	Quadratic	0.99
Chrysin	255.1	153.0	22.53	40	100	Positive	0.25 - 18	Quadratic	0.99
Nordihydroguaiaretic acid	303.0	193.1	22.91	10	100	Positive	0.25 - 18	Quadratic	0.99
Kaempferide	301.0	258.2	24.05	20	100	Positive	0.25 - 18	Quadratic	0.99
Emodin	269.0	225.0	27.29	20	150	Negative	0.25 - 18	Quadratic	0.99
Chrysophanol	255.1	153.0	30.89	40	100	Positive	0.25 - 18	Quadratic	0.99

**Figure 2:**
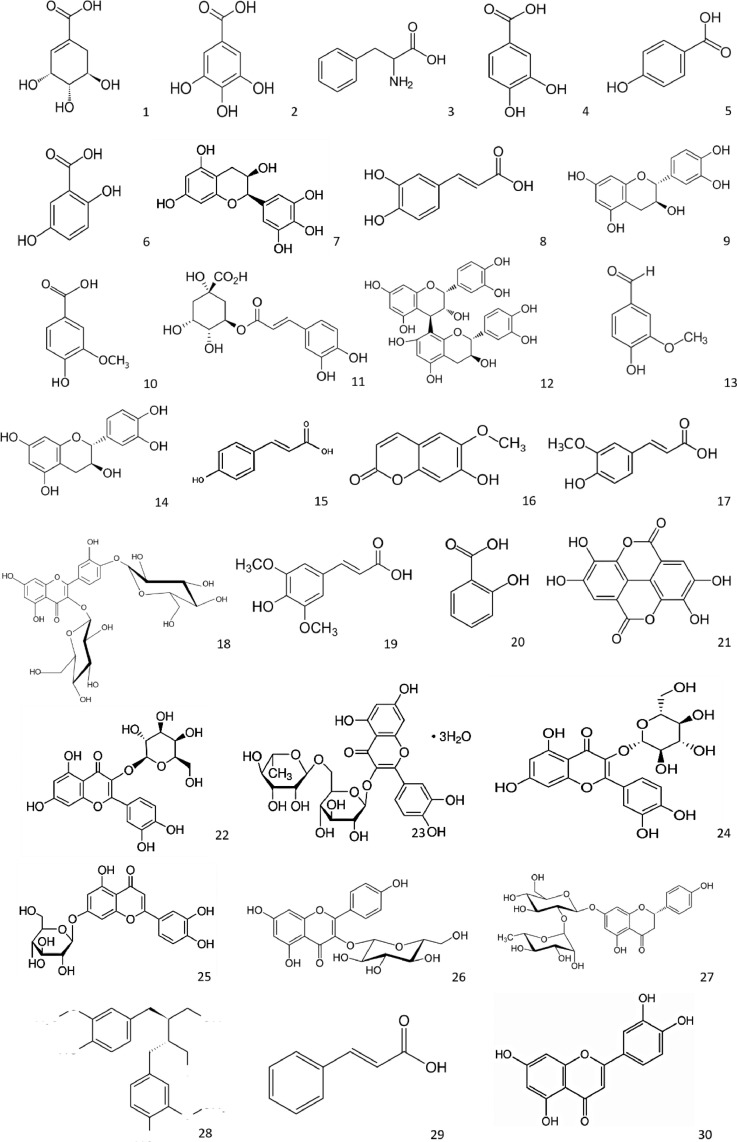
Chemical structure of the phenolic compounds analyzed in extracts of *Acer negundo*. 1) Shikimic acid; 2) Gallic acid; 3) L-phenylalanine; 4) Protocatechuic acid; 5) 4-Hydroxybenzoic acid; 6) Gentisic acid; 7) (-)-Epigallocatechin; 8) Caffeic acid; 9) (+)-Catechin; 10) Vanillic acid; 11) Chlorogenic acid; 12) Procyanidin B2; 13) Vanillin; 14) (-)-Epicatechin; 15) 4-Coumaric acid; 16) Scopoletin; 17) Ferulic acid; 18) Quercetin-3,4´-di-O-glucoside; 19) Sinapic acid; 20) Salicylic acid; 21) Ellagic acid; 22) Quercetin-3-D-galactoside; 23) Rutin trihydrate; 24) Quercetin-3-glucoside; 25) Luteolin-7-O-glucoside; 26) Kaempferol-3-O-glucoside; 27) Naringin; 28) Secoisolariciresinol; 29) trans-Cinnamic acid; and 30) Luteolin.

Phenolic compounds are powerful antioxidants [1, 2]. The 30 chemical structures of the analyzed phenolic compounds are presented in the extracts of leaves and stems of *A. negundo* (Fig. 2).

Thirty phenolic compounds were quantified, in leaf extracts there were 30 compounds and in stem extracts there were 25 compounds (Table 2).

**Table 2:** Concentration of phenolic compounds from *A. negundo* leaf and stem extracts.

Phenolic Compound				Leafs		Stems	
		Molecular Formula	Molecular Weight (g/mol)	mg/g MS	Desvest	mg/g MS	Desvest
1	Shikimic acid	C_7_H_10_O_5_	174.15	311.64	11.93	0.00	0.00
2	Gallic acid	C_7_H_6_O_5_	170.12	4.10	0.23	1.86	0.07
3	L-phenylalanine	C_9_H_11_NO_2_	165.19	173.17	3.14	42.84	0.30
4	Protocatechuic acid	C_7_H_6_O_4_	154.12	1.32	0.88	1.82	0.03
5	4-Hydroxybenzoic acid	C_7_H_6_O_3_	138.12	8.84	0.14	2.24	0.06
6	Gentisic acid	C_7_H_6_O_4_	154.12	181.39	4.08	4.21	0.12
7	(-)-Epigallocatechin	C_15_H_14_O_7_	306.27	3.65	0.12	8.20	0.25
8	Caffeic acid	C_9_H_8_O_4_	180.15	1.49	0.03	0.27	0.01
9	4-Hydroxyphenylacetic acid	C_8_H_8_O_3_	152.14	0.00	0.00	0.00	0.00
10	(+)-Catechin	C_15_H_14_O_6_	290.26	5.82	0.05	65.62	1.33
11	Vanillic acid	C_8_H_8_O_4_	168.14	7.36	0.16	3.83	0.04
12	Scopolin	C_16_H_18_O_9_	354.31	0.00	0.00	0.00	0.00
13	Chlorogenic acid	C_16_H_18_O_9_	354.31	9.56	0.25	0.57	0.03
14	Malvin chloride	C_29_H_35_ClO_17_	691.03	0.00	0.00	0.00	0.00
15	Kuromanin chloride	C_21_H_21_ClO_11_	484.84	0.00	0.00	0.00	0.00
16	Procyanidin B2	C_30_H_26_O_12_	578.52	4.94	0.06	12.31	0.20
17	Vanillin	C_8_H_8_O_3_	152.15	6.35	0.04	3.56	0.04
18	Keracyanin chloride	C_27_H_31_ClO_15_	630.98	0.00	0.00	0.00	0.00
19	(-)-Epicatechin	C_15_H_14_O_6_	290.26	10.36	0.08	37.90	0.38
20	Mangiferin	C_19_H_18_O_11_	422.33	0.00	0.00	0.00	0.00
21	4-Coumaric acid	C_9_H_8_O_3_	164.16	5.65	0.12	1.20	0.03
22	Umbelliferone	C_9_H_6_O_3_	162.14	0.00	0.00	0.00	0.00
23	(-)-Gallocatechin gallate	C_22_H_18_O_11_	458.37	0.00	0.00	0.00	0.00
24	Scopoletin	C_10_H_8_O_4_	192.16	95.70	1.18	2.48	0.05
25	Ferulic acid	C_10_H_10_O_4_	194.18	3.98	0.08	0.90	0.03
26	Quercetin-3,4´-di-O-glucoside	C_27_H_30_O_17_	626.40	33.32	0.51	0.22	0.03
27	Cyanidin	C_15_H_11_O_6_	287.24	0.00	0.00	0.00	0.00
28	3-Coumaric acid	C_9_H_8_O_3_	164.16	0.00	0.00	0.00	0.00
29	Sinapic acid	C_11_H_12_O_5_	224.21	1.45	0.01	0.28	0.02
30	Salicylic acid	C_7_H_6_O_3_	138.12	32.01	1.07	5.97	0.11
31	Ellagic acid	C_14_H_6_O_8_	302.19	173.51	14.40	0.00	0.00
32	(-)-Epicatechin Gallate	C_22_H_18_O_10_	442.37	0.00	0.00	0.00	0.00
33	Myricitrin	C_21_H_20_O_12_	464.37	0.00	0.00	0.00	0.00
34	Pelargonidin chloride	C_15_H_11_ClO_5_	306.70	0.00	0.00	0.00	0.00
35	Quercetin-3-D-galactoside	C_21_H_20_O_12_	464.38	1557.66	25.93	99.68	1.18
36	Rutin trihydrate	C_27_H_30_O_16_ • 3H_2_O	664.56	1776.18	7.54	134.12	1.13
37	Quercetin-3-glucoside	C_21_H_20_O_12_	464.38	1910.18	27.08	81.27	0.84
38	Luteolin-7-O-glucoside	C_21_H_20_O_11_	448.38	264.11	5.34	0.00	0.00
39	p-Anisic acid	C_8_H_8_O_3_	152.14	0.00	0.00	0.00	0.00
40	Malvidin chloride	C_17_H_15_ClO_7_	366.75	0.00	0.00	0.00	0.00
41	2,4-Dimethoxy-6-methylbenzoic acid	C_10_H_12_O_4_	196.20	0.00	0.00	0.00	0.00
42	Penta-O-galloyl-β-D-glucose hydrate	C_41_H_32_O_26_ • xH_2_O	940.68	0.00	0.00	0.00	0.00
43	Kaempferol-3-O-glucoside	C_21_H_20_O_11_	448.37	4238.41	27.55	34.87	0.45
44	Quercitrin	C_21_H_20_O_11_	448.38	0.00	0.00	0.00	0.00
45	Myricetin	C_15_H_10_O_8_	318.24	0.00	0.00	0.00	0.00
46	Naringin	C_27_H_32_O_14_	580.54	9.60	0.30	0.00	0.00
47	trans-Resveratrol	C_14_H_12_O_3_	228,25	0.00	0.00	0.00	0.00
48	Rosmarinic acid	C_18_H_16_O_8_	360,31	0.00	0.00	0.00	0.00
49	Hesperidin	C_28_H_34_O_15_	610,18	0.00	0.00	0.00	0.00
50	Secoisolariciresinol	C_20_H_26_O_6_	362.17	16.18	0.15	0.58	0.06
51	Phloridzin	C_21_H_24_O_10_	436.413	0.00	0.00	0.00	0.00
52	trans-Cinnamic acid	C_9_H_8_O_2_	148.16	0.44	0.01	0.32	0.01
53	Psoralen	C_11_H_6_O_3_	186.16	0.00	0.00	0.00	0.00
54	Quercetin	C_15_H_10_O_7_	302,236	0.00	0.00	0.00	0.00
55	Luteolin	C_15_H_10_O_6_	286.24	41.27	0.94	0.00	0.00
56	Cirsimarin	C_23_H_24_O_11_	476.4	0.00	0.00	0.00	0.00
57	Angelicin	C_11_H_6_O_3_	186.166	0.00	0.00	0.00	0.00
58	Naringenin	C_15_H_12_O_5_	272,25	0.00	0.00	0.00	0.00
59	Apigenin	C_15_H_10_O_5_	270.05	0.00	0.00	0.00	0.00
60	Citropten	C_11_H_10_O_4_	206.19	0.00	0.00	0.00	0.00

**Figure 3:**
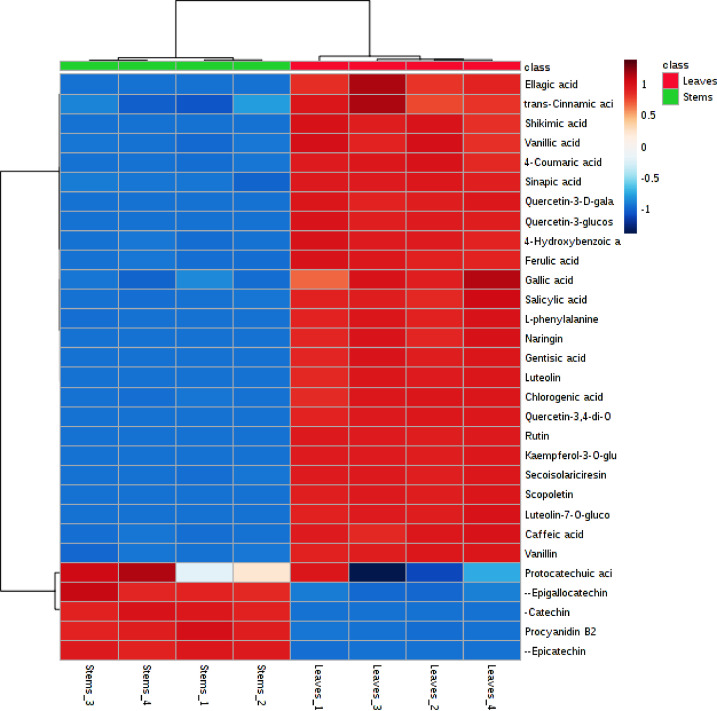
Clustering result shown as heatmap (distance measure using euclidean, and clustering algorithm using ward.D).

Fig. 3 shows a heat map of differential metabolites found by metabolomic analysis. The blue color represents the decreasing trend, the red represents an increasing trend.

**Figure 4:**
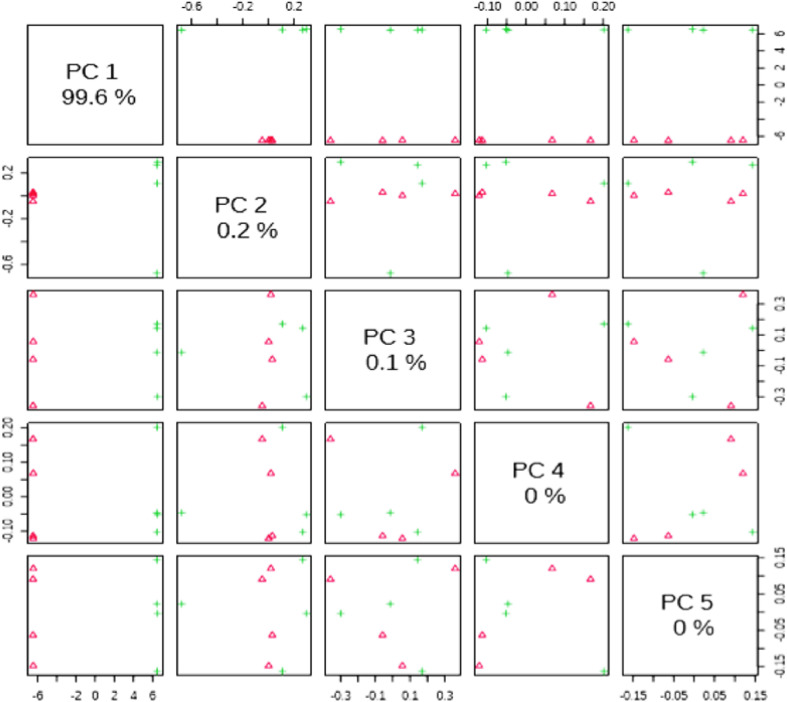
Pairwise score plots between the selected PCs.

Fig. 4 shows the paired scorecards between the selected main components (PCs). The explained variance of each PC is shown in the corresponding diagonal cell.

**Figure 5:**
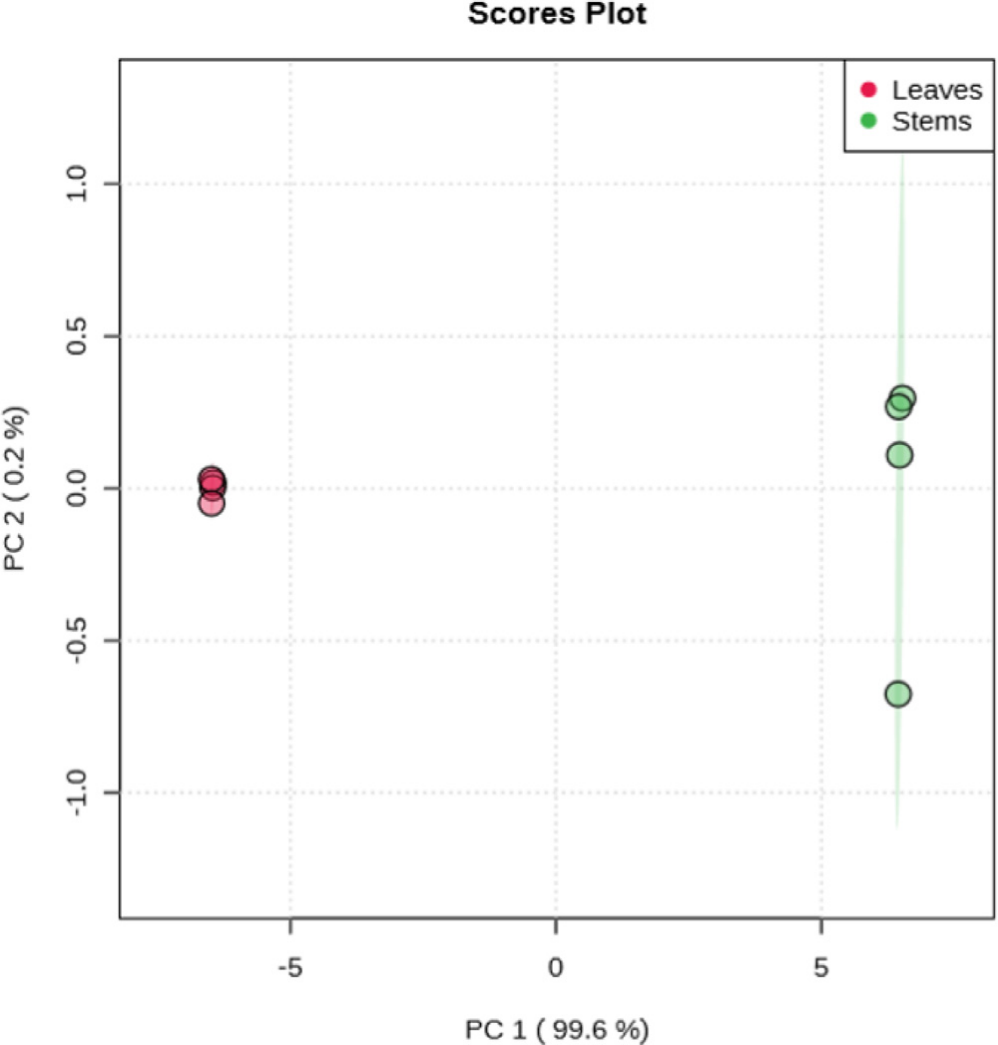
Scores plot between the selected PCs.

Fig. 5 shows a score chart between the selected main components (PCs). The variations explained are shown in brackets.

**Figure 6:**
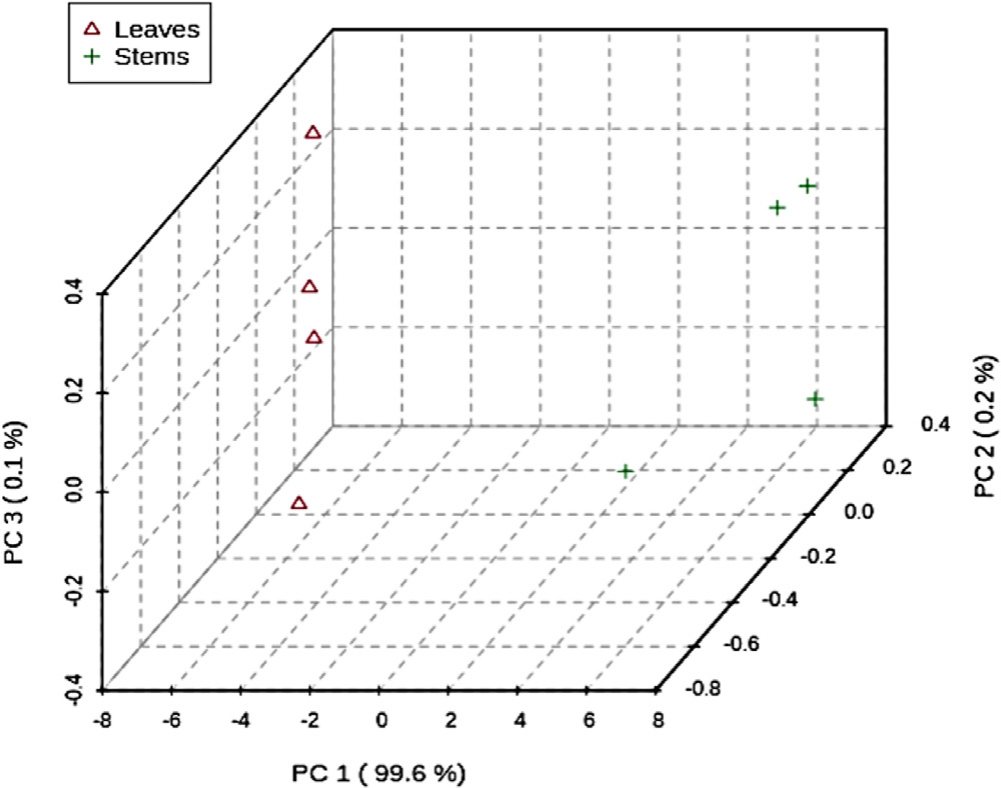
3D score plot between the selected PCs.

In Fig. 6 a 3D score plot is shown between the selected main components (PCs). The explained variations are shown in brackets.

**Figure 7:**
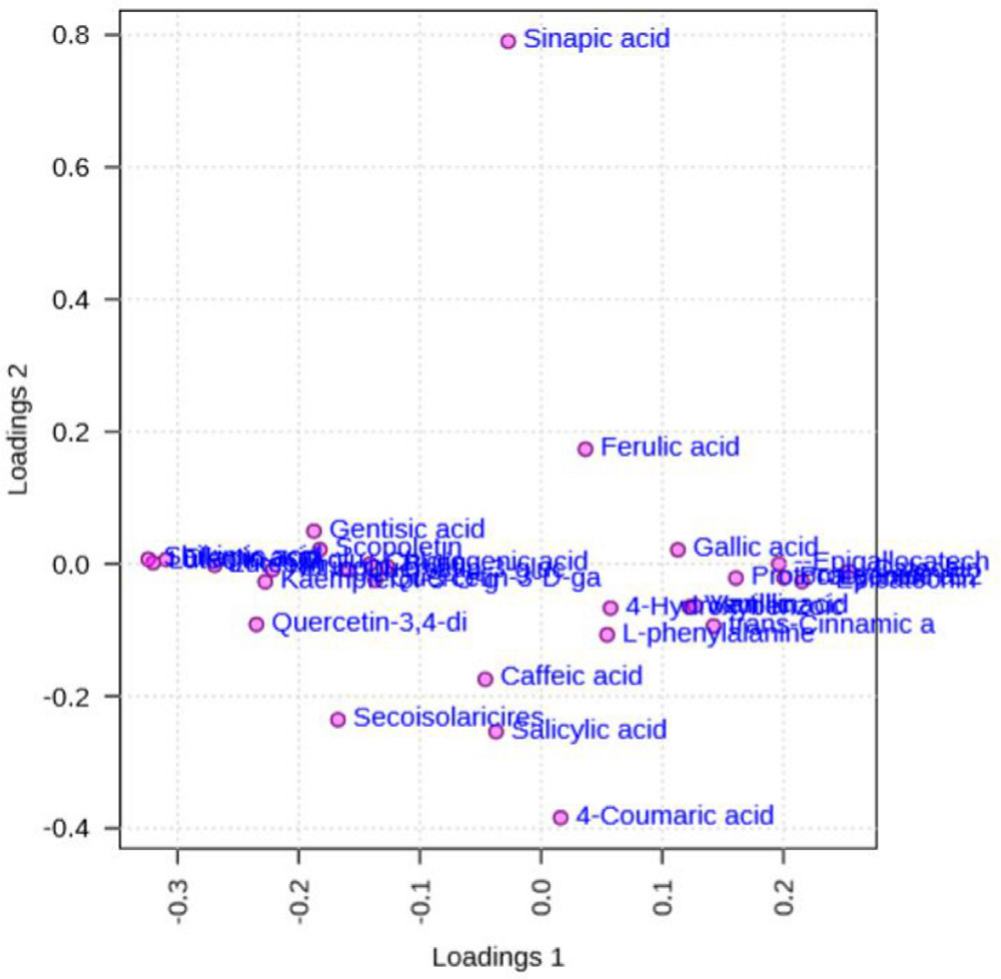
Loadings plot for the selected PCs.

Fig. 7 shows a load plot for the selected main components (PCs).

**Figure 8:**
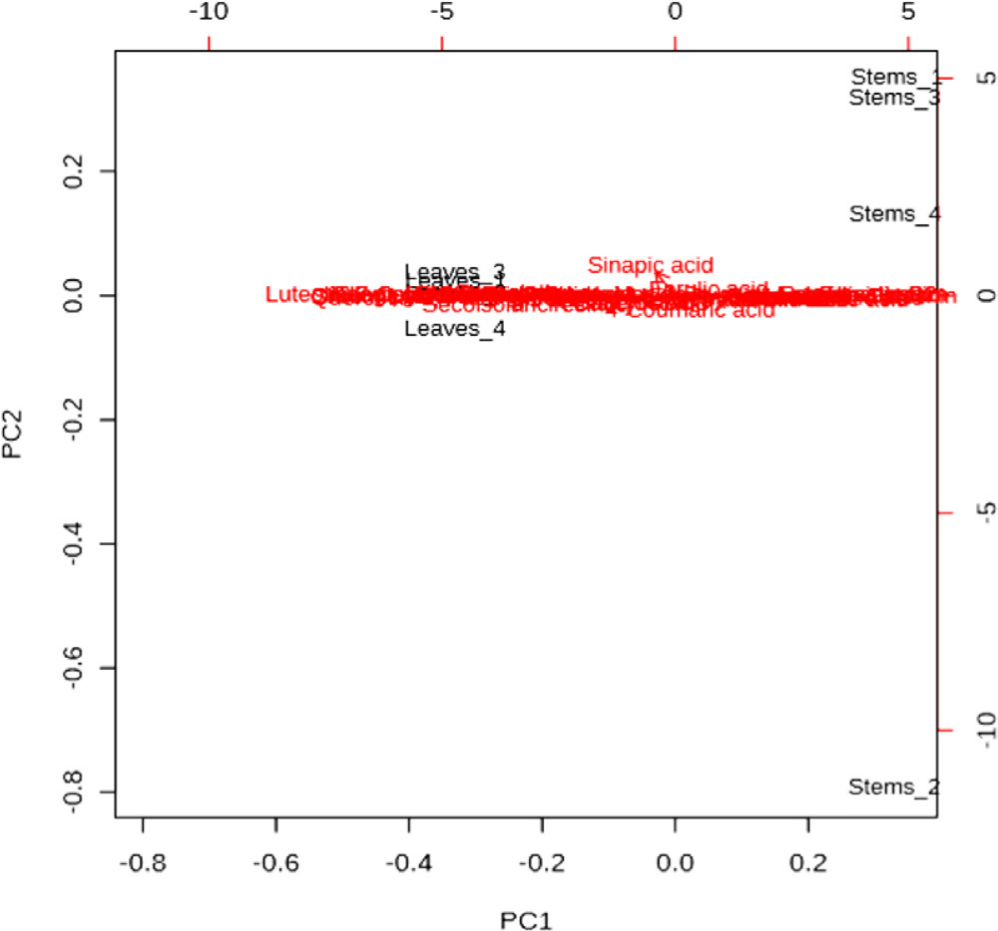
PCA biplot between the selected PCs.

Fig. 8 shows a biplot of the main components among the selected PCs.

## 2 Experimental Design, Materials, and Methods

### 2.1 Extraction data acquisition

The samples come from an *Acer negundo* mother tree free of pests and diseases. Ten leaf and stem samples of *A. negundo* were collected and then subjected to a dehydration process that required three days (72 h) a temperature of 50 °C. Then 100 mg of dry matter was dissolved in 100 mL of 80% ethanol. The mixture was filtered using Whatman No. 1 filter paper. To obtain the raw extracts, a rotary evaporator was used.

### 2.2 Identification and quantification of phenolic compounds

The identification and quantification of phenolic compounds was performed basically as it was previously reported in Juárez-Trujillo *et al*., 2018 [3] and Monribot *et al*., 2019 [4]. The equipment used was a UPLC coupled to a triple quadrupole mass spectrometer. The equipment was injected with 2 µL of ethanolic extract from leaves and 2 µL of ethanolic extract from stems.

### 2.3 Sample preparation

Samples were filtered with 0.5 μm PTFE membranes and placed in 2 mL UPLC vials.

### 2.4 Chromatographic conditions

The data were obtained with a 1290 infinity Agilent ultrahigh resolution liquid chromatograph coupled to a 6460 Agilent triple quadrupole mass spectrometer. The mobile phases were water with 0.1% of formic acid (A) and acetonitrile with 0.1% formic acid (B), both in MS grade. The gradient elution profile is presented in the Table 3.

**Table 3:** 

Time (min)	Solution A (%)	Solution B (%)
0	99	1
30	50	50
35	1	99
39	1	99
40	99	1
45	99	1

The flow was 0.3 mL/min. The injection volume was 2 µL. The column was a Waters, BEH, 2.1 × 50 mm, 1.7 Microns. The column temperature was 40 °C.

### 2.5 Mass spectrometry conditions

The conditions of mass spectrometry is presented in the Table 4.

**Table 4:** 

Parameter	Value
Gas Temp	300 ^o^C
Gas Flow	5 L/min
Nebulizer	45 psi
Sheath Gas Temp	250 ^o^C
Sheath Gas Flow	11 L/min
Capillary voltage (positive and negative)	3500 V
Nozzle voltage (positive and negative)	500 V

## Declaration of Competing Interest

The authors declare that they have no known competing financial interests or personal relationships which have, or could be perceived to have, influenced the work reported in this paper.

**Supplementary materials**

Supplementary material associated with this article can be found, in the online version, at http://dx.doi.org/10.17632/hhp8z52n9t.2 (Mendeley Data).
